# Evidence supporting a role for N^ε^-(3-formyl-3,4-dehydropiperidino)lysine accumulation in Müller glia dysfunction and death in diabetic retinopathy

**Published:** 2010-12-02

**Authors:** Phaik Har Yong, Hongliang Zong, Reinhold J. Medina, G. Astrid Limb, Koji Uchida, Alan W. Stitt, Tim M. Curtis

**Affiliations:** 1 Centre for Vision and Vascular Science, The Queen's University of Belfast, Institute of Clinical Sciences, The Royal Victoria Hospital, Grosvenor Road, Belfast, Northern Ireland; 2 Institute of Ophthalmology, University College London, UK; 3 Department of Food and Biodynamics, Nagoya University, Japan

## Abstract

**Purpose:**

Recent evidence suggests that neuroglial dysfunction and degeneration contributes to the etiology and progression of diabetic retinopathy. Advanced lipoxidation end products (ALEs) have been implicated in the pathology of various diseases, including diabetes and several neurodegenerative disorders. The purpose of the present study was to investigate the possible link between the accumulation of ALEs and neuroretinal changes in diabetic retinopathy.

**Methods:**

Retinal sections obtained from diabetic rats and age-matched controls were processed for immunohistochemistry using antibodies against several well defined ALEs. In vitro experiments were also performed using a human Müller (Moorfields/Institute of Ophthalmology-Müller 1 [MIO-M1]) glia cell line. Western blot analysis was used to measure the accumulation of the acrolein-derived ALE adduct Nε-(3-formyl-3,4-dehydropiperidino)lysine (FDP-lysine) in Müller cells preincubated with FDP-lysine-modified human serum albumin (FDP-lysine-HSA). Responses of Müller cells to FDP-lysine accumulation were investigated by analyzing changes in the protein expression of heme oxygenase-1 (HO-1), glial fibrillary acidic protein (GFAP), and the inwardly rectifying potassium channel Kir4.1. In addition, mRNA expression levels of vascular endothelial growth factor (VEGF), interleukin-6 (IL-6), and tumor necrosis factor-α (TNFα) were determined by reverse transcriptase PCR (RT–PCR). Apoptotic cell death was evaluated by fluorescence-activated cell sorting (FACS) analysis after staining with fluorescein isothiocyanate (FITC)-labeled annexin V and propidium iodide.

**Results:**

No significant differences in the levels of malondialdehyde-, 4-hydroxy-2-nonenal-, and 4-hydroxyhexenal-derived ALEs were evident between control and diabetic retinas after 4 months of diabetes. By contrast, FDP-lysine immunoreactivity was markedly increased in the Müller glia of diabetic rats. Time-course studies revealed that FDP-lysine initially accumulated within Müller glial end feet after only a few months of diabetes and thereafter spread distally throughout their inner radial processes. Exposure of human Müller glia to FDP-lysine-HSA led to a concentration-dependent accumulation of FDP-lysine-modified proteins across a broad molecular mass range. FDP-lysine accumulation was associated with the induction of HO-1, no change in GFAP, a decrease in protein levels of the potassium channel subunit Kir4.1, and upregulation of transcripts for VEGF, IL-6, and TNF-α. Incubation of Müller glia with FDP-lysine-HSA also caused apoptosis at high concentrations.

**Conclusions:**

Collectively, these data strongly suggest that FDP-lysine accumulation could be a major factor contributing to the Müller glial abnormalities occurring in the early stages of diabetic retinopathy.

## Introduction

Diabetic retinopathy is a major cause of vision loss and blindness in people of working age in developed countries [1]. The retinal vasculature is central to the development of diabetic retinopathy, but there is accumulating evidence that neuroretinal function is also compromised during this disease, often before overt vessel changes [2,3]. For example, deficits in visual functioning, such as loss of color vision [4,5], contrast sensitivity [6], and abnormalities in the electroretinogram [7], have been documented in patients shortly after the diagnosis of diabetes and before the detection of clinically evident vascular retinopathy. Early neuronal changes are also apparent in the retinas of experimental rodent models of diabetes, including neurophysiological defects similar to those described in human diabetes [8]. Because neuroretinal changes occur at an early stage of the disease process, it has been proposed that they may play a causative or contributory role in the initiation and progression of the vascular pathology associated with diabetic retinopathy [2,3,9].

Müller cells are the principal glia of the retina. They span the entire thickness of the retina from the inner limiting membrane to the photoreceptor layer, and their processes make contact with most neural cells [10]. They also form end feet on both large vessels and capillaries in the inner and outer retinal vessel beds [11]. Müller glia are vital for maintaining normal neuronal and vascular function in the retina. In addition to their mechanical role in stabilizing the retinal architecture, Müller glia are involved in regulating retinal glucose metabolism, modulating the ionic and molecular composition of the retinal microenvironment, altering blood flow to match the local metabolic needs of the retina, and contributing to the maintenance of the inner blood–retinal barrier [12]. Given their importance in preserving neurovascular function in the retina, there is a strong rationale for exploring Müller glia dysfunction within the context of diabetic retinopathy.

Several studies over the past two decades have provided evidence that Müller glia are adversely affected early in the course of diabetes. Müller glia in both human and experimental diabetes acquire a “reactive” phenotype characterized by cellular hyperplasia [13] and upregulation of glial fibrillary acidic protein (GFAP) [13-15]. In diabetic animals, these gliotic changes are accompanied by several dysfunctional responses, including alterations in their capacity to regulate potassium and glutamate in the extracellular space [14,16,17], accumulation of γ-aminobutyric acid [18], upregulation of pro-inflammatory cytokines [19], and increased expression of angiogenic growth factors, such as vascular endothelial growth factor (VEGF) [20] and insulin-like growth factor (IGF)-1 [21]. Diabetes has also been reported to accelerate death of Müller glia through activation of apoptotic signaling cascades [22]. Although much progress has been made toward characterizing the nature of Müller glial abnormalities in the diabetic retina, the specific mechanisms involved remain poorly understood.

Oxidative stress is strongly implicated in the development of diabetic retinopathy [23]. A possible pathway through which oxidative stress may contribute to retinal cell dysfunction and degenerative changes during diabetes is by promoting the formation of advanced lipoxidation end products (ALEs) [24,25]. ALEs form through the lipoperoxidative production of reactive aldehyde species, such as malondialdehyde (MDA), 4-hydroxy-2-nonenal (HNE), 4-hydroxyhexenal (HHE), and acrolein (ACR), which readily form stable adducts on proteins [26]. These adducts include N^ε^-(2-propenal)lysine and dihydropyridine-type adducts (DHP-lysine; MDA-derived), hemiacetal and pyrrole adducts (HNE- and HHE-derived), and β-substituted propanal adducts and N^ε^-(3-formyl-3,4-dehydropiperidino)lysine (FDP-lysine; ACR-derived) [26]. Such ALEs may induce a variety of cytopathological effects, including cross-linking of cell-surface proteins, inactivation of enzymes, and stimulation of pro-inflammatory and pro-apoptotic signaling pathways [27,28], and they have been identified as pathogenic agents in cardiovascular and neurodegenerative disorders [29]. The pathogenic significance of ALEs in diabetic retinopathy is less well established, although we have recently demonstrated that hemoglobin levels of the ACR-derived ALE, FDP-lysine, are associated with the severity of retinopathy in type 1 and type 2 diabetic patients [25].

In an effort to advance our understanding of the possible contribution of ALE formation in the pathogenesis of diabetic retinopathy, we have performed immunohistochemistry studies using a panel of specific antibodies to assess the accumulation of DHP-lysine, 4-HNE-histidine, 4-HHE-histidine, and FDP-lysine in the retina during early experimental diabetes. Here we report that FDP-lysine selectively and progressively accumulates in retinal Müller glia in experimental diabetes. Complementary in vitro studies demonstrate the effects of FDP-lysine-modified protein on the function and viability of retinal Müller glia. The data indicate that FDP-lysine accumulation may play a key role in mediating Müller glia dysfunction during diabetic retinopathy.

## Methods

### Diabetic studies

#### Experimental animals

Male Sprague-Dawley rats (150–200 g; Harlan Laboratories, Horst, Netherlands) were rendered diabetic by a single intraperitoneal injection of streptozotocin (STZ; 65mg/kg; Sigma, Poole, UK) dissolved in 20 mM/l fresh citrate buffer, pH 4.6. After 1 week, the rats were weighed and those with plasma glucose concentrations >15 mM/l (Ascensia Esprit 2 blood glucose meter, Bayer Diagnostics Europe Ltd, Dublin, Ireland) were included in the study as diabetic. Nondiabetic animals were sham injected with citrate buffer only. Animals were killed by CO_2_ inhalation followed by cervical dislocation 1, 2, 3, or 4 months post injection of STZ or citrate buffer. Animals were reweighed, and blood was collected from the tail vein into heparinized tubes for measurement of percentage levels of glycosylated hemoglobin (GHb; Helena Biosciences, Gateshead, UK) immediately before sacrifice. The number of animals used at each time point in the study is summarized in Table 1. All procedures with animals were approved by The Queen’s University of Belfast Animal Ethics Committee and were performed in accordance with the Guide for the Care and Use of Laboratory Animals published by the US National Institutes of Health (NIH Publication No. 85–23, revised 1996) and the UK Animals (Scientific Procedures) Act, 1986.

**Table 1:** Weight and glycosylated hemoglobin levels of the experimental animals.

**Group**	**Months of diabetes**	**n**	**Bodyweight (g)**	**GHb (%)**
Control	na	9	337±3.9	5.3±0.1
STZ	1	9	238±8.7	20.8±0.8
Control	na	10	419±6.8	6.4±0.3
STZ	2	6	298±15.8	19.5±1.3
Control	na	10	438±8.2	6.4±0.2
STZ	3	10	286±12.5	20±1
Control	na	10	431±9.4	6.4±0.1
STZ	4	9	287±9.4	18.9±1.2

Abbreviations: STZ represents streptozotocin-induced diabetic rats; n represents number of rats per experimental group.

#### Antibodies and advanced lipoxidation end product-modified proteins

Mouse monoclonal anti-FDP-lysine (mAb5F6) and anti-4-HNE (mAbR310) antibodies were prepared as previously described [30,31]. For the detection of HHE- and MDA-modified proteins, monoclonal anti-4-HHE (clone HHE53) and anti-MDA (clone 1F83) antibodies were purchased from the Japan Institute for the Control of Aging (Shizuoka, Japan). FDP-lysine-, 4-HNE-, and 4-HHE-modified human serum albumin (HSA) were prepared by reacting 10 mg/ml HSA (Sigma, Poole, UK) with 10 mM ACR (Sigma), 4-HNE or 4-HHE (Enzo Life Sciences [UK] Ltd, Exeter, UK) in 50 mM sodium phosphate buffer (PBS; 14 mM NaH_2_PO_4_, 36 mM Na_2_HPO_4_, pH 7.2) at 37 °C for 24 h in the dark. Unbound aldehyde was removed by 72 h dialysis at 4 °C against 50 mM PBS with 10 kDa cut-off tubing (Thermo Fisher Scientific, Loughborough, UK). The resulting aldehyde-modified HSA samples were then filter sterilized with a 0.2-μm filter (Millipore, Watford, UK) before use. MDA-modified BSA (BSA) was purchased from Abcam (Cambridge, UK). Polyclonal antibodies against heme oxygenase 1 (HO-1), GFAP, and Kir4.1 were obtained from Abcam, Dako (Ely, UK), and Caltag-Medsystems Ltd (Buckingham, UK), respectively.

#### Dot blot analysis of antibody specificity

Dot blot analysis was used to test the specificity of the monoclonal anti-ALE antibodies. Polyvinylidene fluoride (PVDF) membranes (Westran®, Roche Diagnostics Limited, Burgess Hill, West Sussex, UK) were dipped in 100% methanol, rinsed with water, then soaked in Tris-buffered saline with Tween-20 (TTBS; containing 100 mM Tris-HCl, 150 mM NaCl, 0.1% Tween-20 [v/v], pH 7.5) for 5 min. Five microliters of each ALE-modified protein, containing 12 μg protein, were then spotted onto the PVDF membrane and allowed to slowly air dry overnight. Membranes were washed, and nonspecific protein-binding sites blocked with 5% nonfat dry milk for 2 h at 21 °C. Membranes were washed again and incubated overnight at 4 °C with primary antibody diluted 1:100 (anti-4-HNE-, anti-4-HHE, or anti-MDA antibodies) or 1:1,000 (anti-FDP-lysine antibody) with TTBS containing 2% nonfat dry milk. After extensive washing with TTBS, membranes were exposed for 1 h at 21 °C to peroxidase-labeled goat antimouse immunoglobulin (IgG) antibody (diluted 1:200; Sigma). Membranes were washed again and the blots developed using the Immobilon Western chemiluminescent horseradish peroxidase substrate (Millipore) and detected by an Autochemi^TM^ system (UltraViolet Products Ltd, Cambridge, UK).

#### Immunohistochemistry

Eyes were enucleated, hemisected along the ora serrata, and the vitreous humor removed. Eyecups were immersion fixed in 4% (w/v) paraformaldehyde for 30 min, washed in PBS, and the retinas detached. Fixed retinas were cryoprotected, embedded in Tissue-Tek OCT compound (Sakura Finetek, Torrance, CA), snap frozen in liquid nitrogen-cooled isopentane, and 12-μm cryosections prepared.

Cryosections were mounted onto slides, rinsed in PBS, and nonspecific binding sites blocked using 10% normal donkey serum and 0.5% Triton X-100 in PBS. The slides were then rinsed in PBS and incubated with the primary antibody diluted in 10% normal goat serum, 0.3% Triton X-100, and 0.1% NaN_3_ for 24 h at 4 °C. Primary antibodies were used at the following dilutions: anti-4-HNE, anti-4HHE, and anti-MDA, 1:100; anti-FDP-lysine and anti-GFAP, 1:1,000. Sections were rinsed thoroughly to remove unbound primary antibody and incubated in an appropriate fluorescent-conjugated secondary antibody (Alexa fluor^488^ donkey antimouse IgG or donkey antirabbit IgG; Invitrogen, Paisley, UK), diluted 1:200 in 10% donkey serum in PBS, for 1 h at room temperature. Slides were mounted in Vectashield antifade (Vector Laboratories, Peterborough, UK) containing 5 μg/ml propidium iodide. Multiple immunolabeling was performed by simultaneous incubation of sections with anti-FDP-lysine and anti-GFAP, and the sections subsequently washed and incubated in secondary antibodies Alexa fluor^488^ donkey antimouse IgG and Alexa fluor^568^ donkey antirabbit IgG. For these slides Vectashield in the presence of 1.5 μg/ml DAPI nuclear stain was used as the mounting media. Appropriate negative and pre-absorption controls were also performed.

Fluorescence was visualized by using a Nikon TE-2000 C1 confocal system (Nikon Ltd, Kingston upon Thames, UK). Images were obtained from three areas of the retina: inferior central (0 to 1 mm below the optic nerve head) and inferior peripheral and superior peripheral (0 to 2 mm from the inferior or superior peripheral edge of the retina, respectively) regions. Measurements of immunofluorescence intensity were performed using ImageJ software (Wayne Rasband, NIH, Bethesda, MD) as previously described [32]. In some sections it was not possible to identify precisely the boundaries between the inner limiting membrane (ILM) and nerve fiber layer (NFL) and the NFL and ganglion cell layer (GCL), and therefore these regions were combined for quantification. Either the left or the right eye were analyzed for each rat. In each experimental protocol, a total of nine tissue sections was imaged per retina (three sections from each of the three designated areas examined), and mean values were calculated for use in subsequent statistical analyses.

### In vitro studies

MIO-M1 Müller glia cells [33] were maintained in Dulbecco’s modified Eagle’s medium supplemented with 10% fetal calf serum and 10 mg/ml primocin. Cells were grown to near confluency, serum starved for 24 h, and then treated with FDP-lysine-HSA at concentrations from 0.05 to 0.5 mg/ml for an additional 24 h. Cells were used from passages 42 to 55. All in vitro experiments were performed in triplicate.

#### Western blot analysis

Western immunoblotting was performed as previously described [34]. Briefly, protein samples of MIO-M1 cells exposed to FDP-lysine-HSA were prepared by scraping the cells off the flask in an extraction buffer solution of PBS containing protease inhibitors (Roche Diagnostics). Protein concentration was determined using the BCA protein assay kit (Thermo Fisher Scientific), and 30 μg of protein was subjected to 10% SDS–PAGE. Western blotting was performed by incubating the PVDF membrane with anti-FDP-lysine (1:10,000), anti-HO-1 (1:1,000), anti-GFAP (1:3,000), or anti-Kir4.1 (1:500) antibodies together with rabbit polyclonal or mouse monoclonal β-actin antibodies (1:3,000 and 1:20,000, respectively; Sigma) at 4 °C overnight. After washing, the membrane was incubated with appropriate IRDye 680 and IRDye 800 goat secondary antibodies against mouse or rabbit IgG (Li-COR, Cambridge, UK) at room temperature for 30 min. Membranes were imaged with an Odyssey Infrared Imaging System (Li-COR). Color images were converted to grayscale, background subtracted, and the integrated density of protein bands determined using ImageJ. Data were normalized to β-actin-loading controls and presented as fold changes compared with untreated cells.

#### RNA isolation and RT–PCR

MIO-M1 RNA was extracted using Tri-Reagent (Sigma), and quality was confirmed by measurement of the A_260_:A_280_ ratio. The Improm-II reverse transcription system (ImProm II, Promega, MSC, Dublin, Ireland) was used to synthesize cDNA, with 1 μg of MIO-M1 RNA and random hexamer primers, according to the manufacturer's instructions. Real-time PCR analysis was performed for quantitative analysis of mRNA expression as previously described [35]. Sequence-specific primers were designed using the program Primer3 to amplify VEGF, interleukin-6 (IL-6), tumor necrosis factor-α (TNF-α), and β-actin (for primer sequence data, see Table 2). Results were normalized to β-actin, and expression was compared to normal levels in untreated cells using the delta Ct method [35].

**Table 2:** Primer sequences for quantitative RT–PCR and GenBank accession numbers.

**Gene**	**Forward primer (5′-3′)**	**Reverse primer (5′-3′)**	**Accession number**
*VEGFA*	CCCACTGAGGAGTCCAACAT	TTTCTTGCGCTTTCGTTTTT	NM_001025366.2
*IL-6*	TACCCCCAGGAGAAGATTCC	TTTTCTGCCAGTGCCTCTTT	NM_000600.3
*TNFα*	AACCTCCTCTCTGCCATCAA	CCAAAGTAGACCTGCCCAGA	NM_000594.2
*β-actin*	AGAGCTACGAGCTGCCTGAC	AGCACTGTGTTGGCGTACAG	NM_001101.3

#### Apoptosis analysis

Apoptosis was analyzed by flow cytometry, using annexin V and propidium iodide according to the manufacturer’s instructions (Annexin V-FITC Apoptosis Detection Kit I; BD Biosciences, Oxford, UK). Briefly, after treatment cells were trypsinized, washed in cold PBS, and resuspended in assay buffer. Annexin V and propidium iodide solution were added to the cell preparations and incubated for 15 min in the dark at room temperature. Binding buffer was then added to each tube, and the samples were analyzed using a FACS Calibur flow cytometer (BD Biosciences) and FlowJo software V7.5.5 (Tree Star, Ashland, OR). For each sample, 20,000 events were acquired. Respective negative controls were used to determine accurate settings for data analysis.

### Data analysis and statistical calculations

Data are presented as means±SEM. Statistical analyses were performed using Prism V4.02 (Graphpad Software, San Diego, CA). Western blot and qRT–PCR data were analyzed before conversion to fold-change units, while data on apoptosis were arcsine transformed before analysis. Normal distribution was assessed by the D’Agostino and Pearson omnibus normality test. For immunohistochemical and apoptosis data, differences among groups were analyzed with a two-way ANOVA followed by Bonferroni’s post hoc test. All other data were analyzed by one-way ANOVA followed by Newman–Keuls multiple comparison. In all figures, significant differences are represented with a single asterisk when the p value is less than 0.05, with two asterisks when the p value is less than 0.01, and three asterisks when the p value is less than 0.001.

## Results

### Animals

The general condition of the STZ-diabetic rats was assessed by measuring bodyweights and GHb (Table 1). All STZ-diabetic animals gained weight during the course of the study, but their growth was significantly reduced as compared to that observed in control animals (p<0.001 for all time points after diabetes induction). Nondiabetic rats had normal mean GHb values ranging between 5.3% and 6.4%, while in diabetic rats the mean GHb levels were threefold to fourfold higher (p<0.001).

### Epitope specificity of anti-ALE antibodies

**Figure 1:**
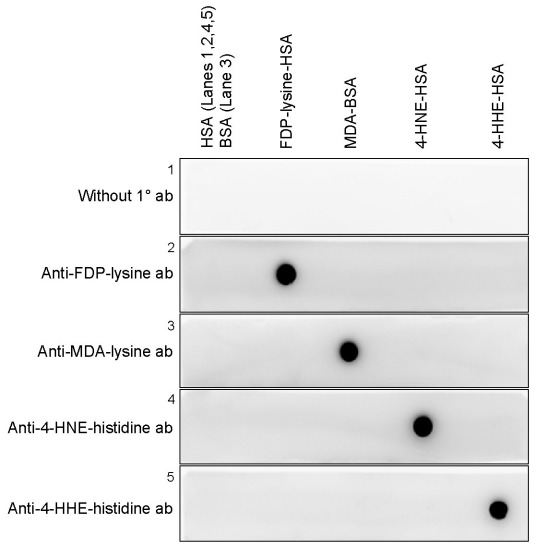
Specificity of the anti-advanced lipoxidation end product antibodies. Representative dot blots showing the specificity of the anti-advanced lipoxidation end product (ALE) monoclonal antibodies to aldehyde-modified proteins. Anti-Nε-(3-formyl-3,4-dehydropiperidino)lysine (FDP-lysine), anti-malondialdehyde (MDA)-lysine, anti-4-hydroxy-2-nonenal (HNE)–histidine, and anti-4-hydroxyhexenal (HHE)-histidine monoclonal antibodies reacted specifically with FDP-lysine human serum albumin (HSA), MDA BSA (BSA), 4-HNE-HSA, and 4-HHE-HSA, respectively.

Prior to immunolocalization studies, the specificities of the anti-ALE antibodies were tested by western dot blot analysis. The reactivity of the antibodies was examined against FDP-lysine-HSA, MDA-BSA, 4-HNE-HSA, and 4-HHE-HSA. Each of the antibodies reacted strongly with its corresponding antigenic protein with no cross-reactivity (Figure 1). No immunoreactivity was detected with the relevant carrier proteins HSA or BSA.

### Selective accumulation of FDP-lysine adducts in retinal Müller glia during diabetes

**Figure 2:**
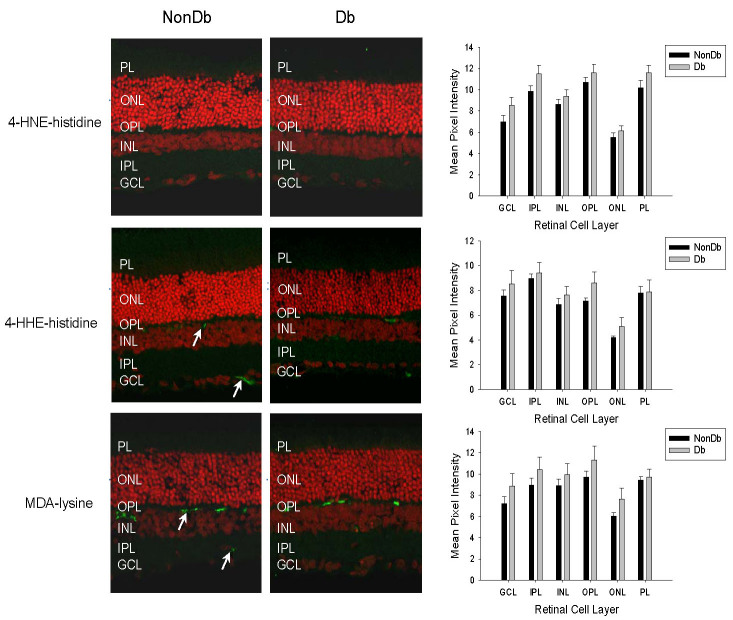
Immunohistochemical staining for 4-hydroxy-2-nonenal (HNE), 4-hydroxyhexenal (HHE), and malondialdehyde (MDA) modified proteins (green fluorescence) in transverse cryosections of retina from nondiabetic (NonDb) and diabetic (Db) rats of 4 months disease duration. Propidium iodide was used to counterstain cell nuclei (red fluorescence). In nondiabetic retina, weak diffuse immunoreactivity for each of these advanced lipoxidation end products (ALEs) was detected, although stronger cytoplasmic staining for 4-HHE and MDA-modified proteins was observed for a small number of cells located within the ganglion cell layer (GCL) and at the outer border of the inner nuclear layer (INL; arrows). Immunolabelled sections from diabetic rats appeared similar to those of nondiabetic rats. When quantified, no significant differences in the intensity of staining for 4-HNE, 4-HHE, and MDA modified proteins was apparent between retinas from the nondiabetic and diabetic animals.

**Figure 3:**
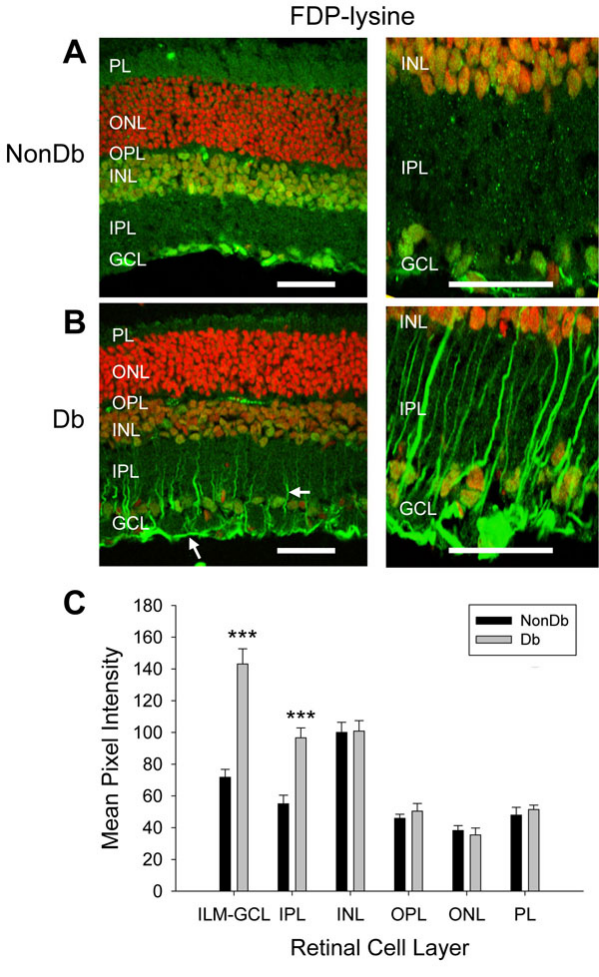
Nε-(3-formyl-3,4-dehydropiperidino)lysine (FDP-lysine) immunoreactivity (green fluorescence) in vertical sections of retina from nondiabetic (NonDb) and diabetic (Db) rats of 4 months disease duration. Nuclei were counterstained with propidium iodide (red fluorescence). **A**: Low and high magnification images of FDP-lysine immunoreactivity in the normal rat retina. Strong immunolabelling was detected in the retinal ganglion cell layer (GCL) and nuclei in the inner nuclear layer (INL). **B**: Low and high power confocal micrographs of FDP-lysine immunoreactivity in diabetic rat retina. Prominent immunolabelling appeared at the ILM and in radial processes in the inner retina (arrows). **C**: Summary data showing that diabetes caused a statistically significant increase in FDP-lysine immunolabelling limited the innermost retinal layers.

We began by examining the distribution and accumulation of ALE adducts in retinal tissues from diabetic rats of 4 months disease duration. Previous work has reported that only very low levels of 4-HNE-, 4-HHE-, and MDA-modified proteins are detectable in retinal tissues from normal control animals [36,37]. Similarly, we found sparse immunoreactivity for these adducts in the retinas of nondiabetic rats (Figure 2). No differences in the intensity or distribution of the staining for 4-HNE-, 4-HHE, and MDA-modified proteins were evident in retinas from diabetic rats (Figure 2; p>0.05 for all cell layers). In the nondiabetic retina, the anti-FDP-lysine antibody strongly stained a population of cells in the GCL and nuclei in the inner nuclear layer (INL, Figure 3A), and diffuse immunoreactivity was present across the other layers of the retina. In diabetic retina there was a marked increase in FDP-lysine immunoreactivity in the inner layers of the retina (Figure 3B,C). In particular, prominent immunolabelling was evident at the ILM and within radial processes extending from the ILM to the INL. This staining pattern corresponded to Müller glia with end feet at the ILM and distal processes traversing the inner plexiform layer (IPL) [10]. Primary antibody omission and pre-absorption controls were completely negative for all ALEs investigated (mean pixel intensities were <1 after background subtraction).

**Figure 4:**
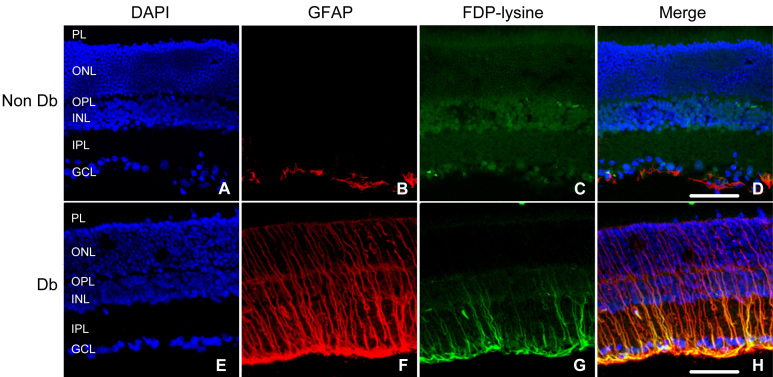
Co-localization of Nε-(3-formyl-3,4-dehydropiperidino)lysine (FDP-lysine) and glial fibrillary acidic protein (GFAP) in the diabetic retina. Retinal sections from nondiabetic (NonDb) and diabetic (Db) rats were labeled with 4',6-diamidino-2-phenylindole (DAPI) nuclear stain (**A** and **E**), GFAP (**B** and **F**), and FDP-lysine (**C** and **G**). In the nondiabetic retina, GFAP immunoreactivity was selectively localized to astrocytes at the inner retinal surface. In diabetes, Müller cells acquired prominent GFAP immunoreactivity within their end feet at the vitreoretinal border and throughout their radial processes spanning from the inner to the outer limiting membranes. **D** and **H**: In merged images, FDP-lysine was found to co-localize strongly with GFAP in the retina of diabetic but not nondiabetic rats. The scale bars indicate 50 μm.

To confirm that FDP-lysine selectively accumulates in retinal Müller glia during diabetes, double-labeling experiments were performed using anti-FDP-lysine and anti-GFAP antibodies (Figure 4). Previous studies have shown that after 4 months of STZ-induced diabetes, retinal GFAP is upregulated in Müller glia and relatively downregulated in astrocytes [38]. In nondiabetic rat retinas, GFAP expression was limited to cells along the inner margin of the retina with a morphology and distribution characteristic of astrocytes. In diabetic retina, intense GFAP immunoreactivity was detected in both the Müller glial end feet and along radial processes extending to the outer limiting membrane. FDP-lysine co-localized with GFAP in the diabetic retina, suggesting that the accumulation of this adduct was largely restricted to Müller glia.

### Spatiotemporal pattern of FDP-lysine accumulation in diabetic Müller cells

**Figure 5:**
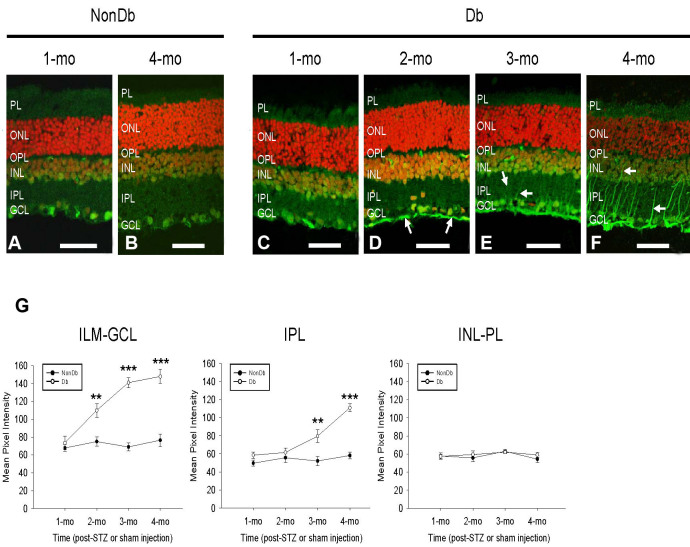
Spatiotemporal characteristics of Nε-(3-formyl-3,4-dehydropiperidino)lysine (FDP-lysine) accumulation in diabetic Müller cells. **A** and **B**: Representative confocal images of FDP-lysine immunoreactivity in retinal sections from non-diabetic rats 1 and 4 months post injection of citrate buffer. **C**-**F**: Immunohistochemisty for FDP-lysine in retinal sections from STZ-diabetic rats 1, 2, 3, and 4 months after the induction of diabetes. The intensity of FDP-lysine staining initially increased in the end feet of Müller cells at the ILM after 2 months of diabetes (**D**, arrows) and subsequently spread throughout their inner processes extending from the ILM to the INL (**E**, **F**, arrows). **G**: Summary data revealed that FDP-lysine immunoreactivity was significantly increased in diabetic retina in regions of the ILM–GCL and IPL, 2 and 3 months following the onset of diabetes, respectively. There was no significant difference in FDP-lysine in the outer retina at any time point between the nondiabetic and diabetic groups. The scale bars refer to 50 μm.

The spatiotemporal pattern of FDP-lysine accumulation in Müller glia during experimental diabetes was investigated over a period of 1 to 4 months disease duration. As shown in Figure 5, no significant changes in FDP-lysine immunoreactivity were evident over the first month of diabetes. At 2 months, FDP-lysine levels were strongly elevated at the ILM, but no detectable increase was apparent in the IPL. By 3 months of diabetes, strong staining for FDP-lysine was not only visible along the ILM, but also in the NFL and GCL. In addition, a small but significant increase in FDP-lysine immunoreactivity was seen in the IPL due to faint staining of radial processes. As described earlier, after 4 months of diabetes, strong positive immunolabelling for FDP-lysine was seen in both the Müller glial end feet at the ILM and their adherent inner processes. No discernible changes in FDP-lysine immunoreactivity were seen in the nondiabetic rat retina over the 4-month time frame. Overall, these results strongly suggest that the accumulation of FDP-lysine-modified proteins in Müller glia is progressive as a function of diabetes duration, with adducts initially localizing to the end feet at the vitreoretinal border and then spreading distally to processes at the outer retina.

### FDP-lysine accumulation mediates Müller glia dysfunction and death in vitro

**Figure 6:**
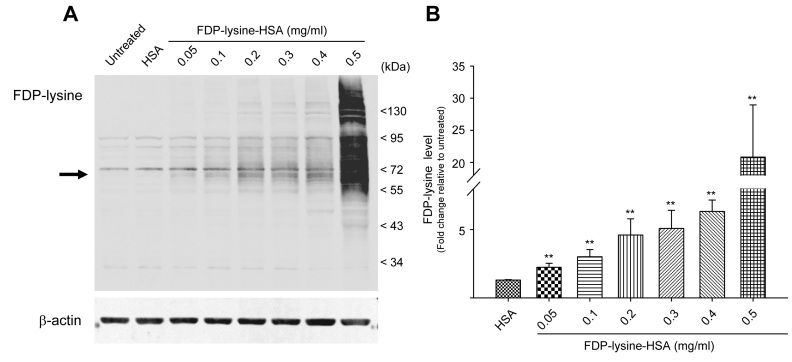
In vitro model of Nε-(3-formyl-3,4-dehydropiperidino)lysine (FDP-lysine) accumulation in Müller cells. **A**: Representative western blot image showing the abundance of FDP-lysine adducts in human Müller (MIO-M1) cells under control conditions and following treatment with increasing concentrations of FDP-lysine human serum albumin (HSA). MIO-M1 cells exposed to FDP-lysine HSA exhibited a concentration-dependent increase in FDP-lysine immunoreactivity across a broad molecular mass range. The arrow indicates the expected size of monomeric FDP-lysine-HSA. **B**: Mean data showing the fold change in FDP-lysine levels for each treatment group relative to untreated cells.

To gain an insight into the pathogenic potential of FDP-lysine in Muller glia, we adopted an in vitro approach. Western blotting for FDP-lysine adducts from both control and FDP-lysine-HSA exposed MIO-M1 cells demonstrated a concentration-dependent increase in the abundance of FDP-lysine protein adducts over a broad molecular mass range (Figure 6). It was notable that a step change in FDP-lysine immunoreactivity occurred at the highest concentration of FDP-lysine-HSA studied (0.5 mg/ml). Cells incubated with FDP-lysine-HSA showed a ~68 kDa band (consistent with FDP-lysine-HSA), which was absent in lysates from control cells (arrow, Figure 6A). In addition, several higher molecular weight bands were evident in cells exposed to concentrations of FDP-lysine-HSA ≥0.2 mg/ml. These data correspond with previous work showing that FDP-lysine is not a stable end product but acts as a reactive intermediate capable of mediating protein–protein cross-linking reactions [39].

**Figure 7:**
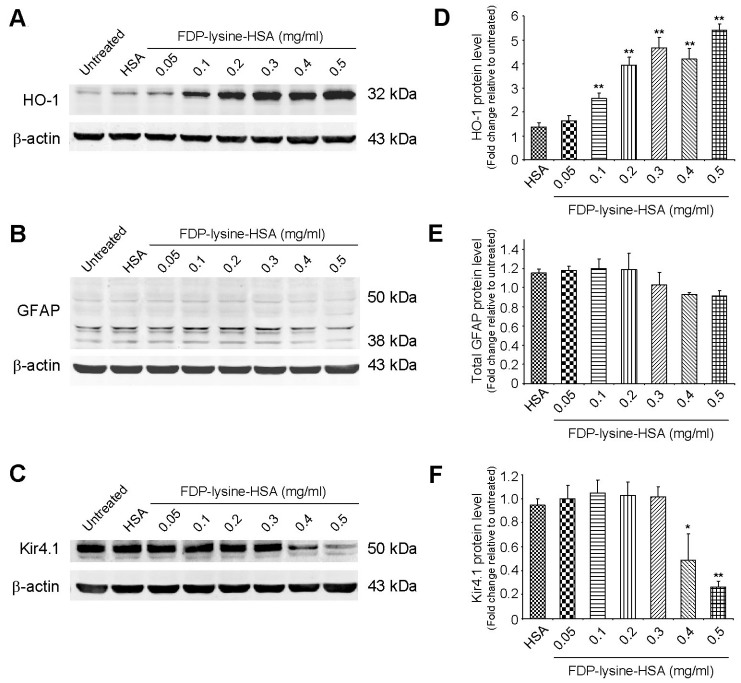
Differential effects of Nε-(3-formyl-3,4-dehydropiperidino)lysine (FDP-lysine) on heme oxygenase 1 (HO-1), glial fibrillary acidic protein (GFAP), and Kir4.1 protein levels. **A**-**F**: Representative western blots and corresponding quantification for HO-1, GFAP, and Kir4.1 protein levels in control and FDP-lysine human serum albumin (HSA)-treated human Muller (MIO-M1) cells. Incubation with FDP-lysine-HSA evoked a concentration-dependent increase in HO-1, no change in total GFAP levels, and a decrease in Kir4.1 protein expression at high treatment concentrations (≥0.4 mg/ml).

Diabetes has been demonstrated to induce Müller cell oxidative stress, as indicated by an increase in the protein expression of the antioxidant enzyme HO-1 [40]. In addition to being generated through oxidative reactions, it has been proposed that FDP-lysine adducts could give rise to oxidative stress via depletion of glutathione within the cell [39]. Treatment of MIO-M1 cells with FDP-lysine-HSA stimulated a concentration-dependent increase in HO-1 protein levels (Figure 7A,D). In western blots of control cell extracts, GFAP appeared as multimeric bands at 38, 42, 43, and 50 kDa (Figure 7B,E), and no significant differences were observed in these bands following FDP-lysine-HSA exposure. Another important alteration that occurs in diabetic Müller cells is a disruption in their ability to buffer extracellular K^+^ levels in the retina [12]. The primary K^+^ conductance in Müller cells is mediated by the inwardly rectifying potassium channel Kir4.1 [12], and there was an appreciable downregulation of Kir4.1 protein levels in MIO-M1 cells treated with high concentrations of FDP-lysine-HSA (≥0.4 mg/ml; Figure 7C,F).

**Figure 8:**
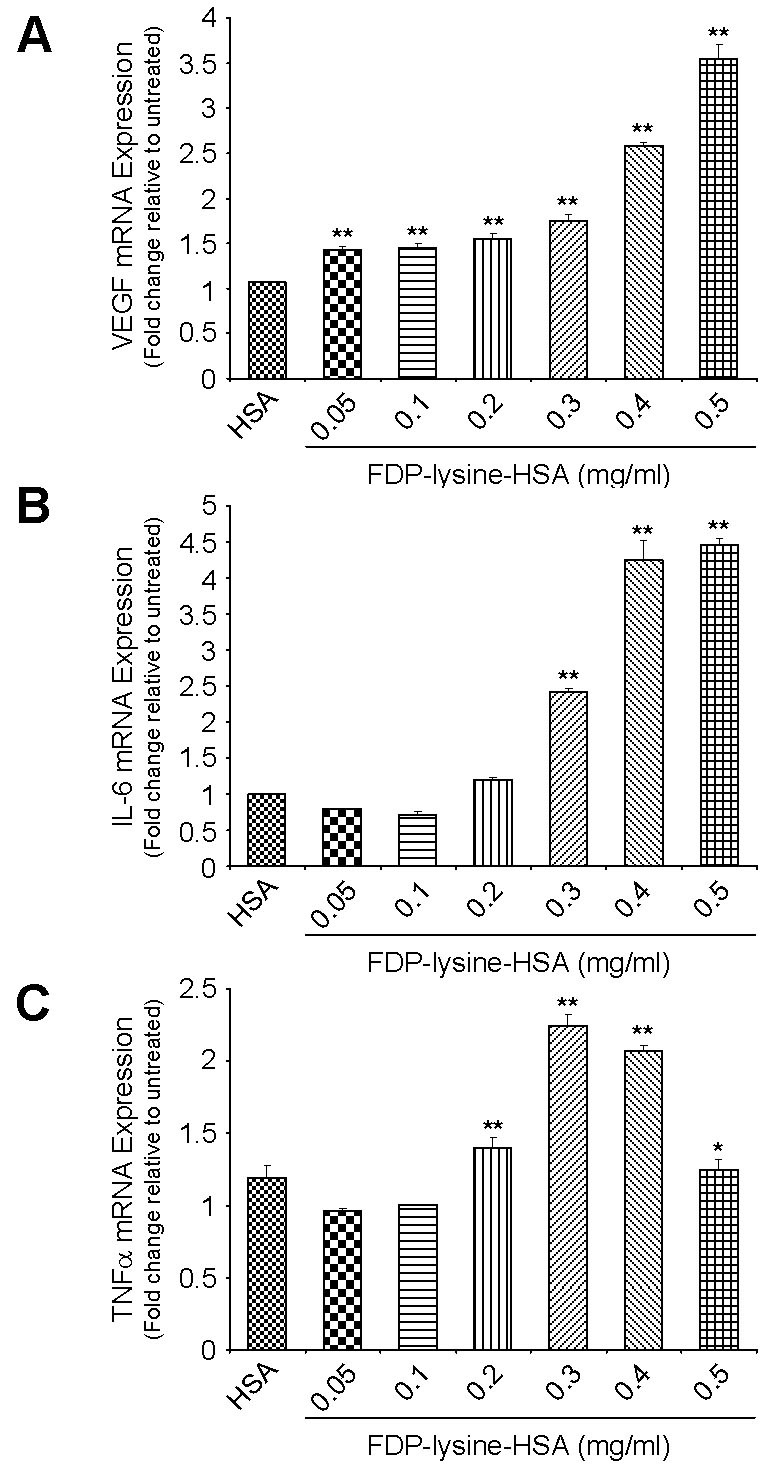
Nε-(3-formyl-3,4-dehydropiperidino)lysine (FDP-lysine) accumulation is associated with changes in angiogenic and inflammatory gene expression. Bar graphs showing the concentration-dependent induction of (**A**) vascular endothelial growth factor (VEGF), (**B**) interleukin-6 (IL-6), and (**C**) tumor necrosis factor-α (TNF-α) mRNAs following exposure of human Muller (MIO-M1) cells to FDP-lysine human serum albumin (HSA). mRNA levels were analyzed by real-time quantitative PCR using gene-specific primers and normalized with β-actin as the internal control.

FDP-lysine-HSA exposure altered mRNA expression levels of angiogenic and inflammatory factors known to be elevated in the vitreous and retina during diabetes. FDP-lysine-HSA led to a concentration-dependent increase in VEGF transcript levels across the full range of concentrations tested (Figure 8A). Similar results were obtained for IL-6, although the threshold concentration for the induction of IL-6 gene expression was higher than that required for VEGF (0.3 mg/ml FDP-lysine-HSA; Figure 8B). FDP-lysine-HSA also elevated TNF-α transcript levels, an effect that increased to a maximum at 0.3 mg/ml and then became less pronounced at higher concentrations (Figure 8C).

**Figure 9:**
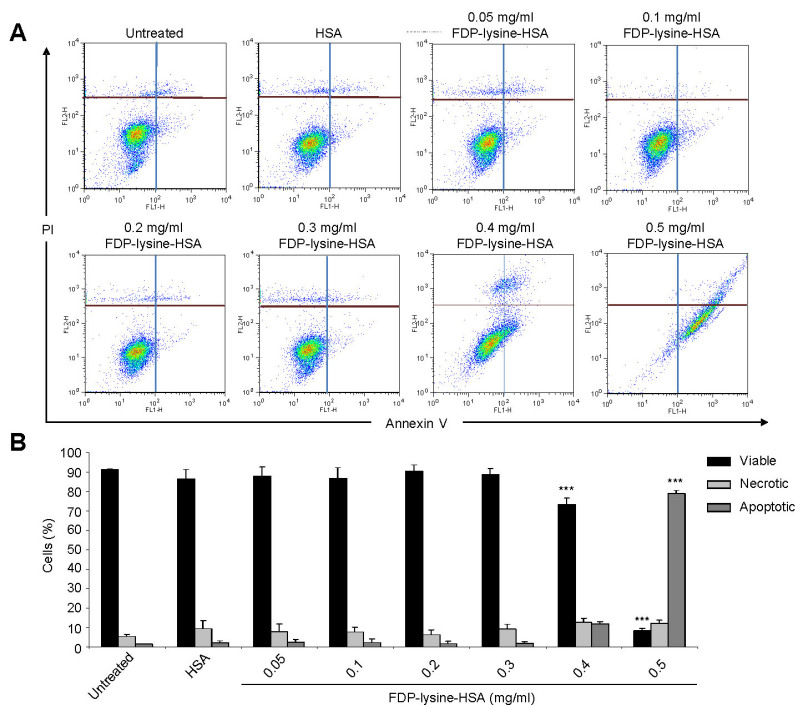
Fluorescence-activated cell sorting (FACS) analysis of apoptosis in human Muller (MIO-M1) cells incubated with Nε-(3-formyl-3,4-dehydropiperidino)lysine (FDP-lysine) human serum albumin (HSA) measured using fluorescein isothiocyanate (FITC)-labeled annexin V and PI. **A**: Representative scatter plots showing the distribution of annexin V and PI staining for control and FDP-lysine-HSA-treated MIO-M1 cells. Cells are classified as “viable” (bottom left), “apoptotic” (bottom right), or “necrotic” (top left and right). Cells exposed to 0.5 mg/ml FDP-lysine HSA showed a distinct shift into the bottom right quadrant, consistent with apoptosis (**B**). Quantitative analysis of the percentage of viable, apoptotic, or necrotic cells by FACS analysis. *** p<0.001 versus untreated cells.

As determined by annexin V labeling, control cells and those treated with concentrations of FDP-lysine-HSA ≤0.3 mg/ml remained viable and were primarily Annexin V and PI negative (Figure 9). A rightward shift in the FACS profile, indicative of apoptosis, was observed for cells treated with >0.4 mg/ml FDP-lysine-HSA (Figure 9).

## Discussion

To the best of our knowledge, this study is the first to address the distribution pattern of ALEs in the diabetic retina. During early experimental diabetes there is a selective accumulation of the acrolein-derived ALE, FDP-lysine, in the Müller glia, whereas no changes were detected in the intensity or distribution of other ALE adducts, including 4-HNE-histidine, 4-HHE-histidine, and MDA-lysine. The accumulation of FDP-lysine begins after only a few months of diabetes and is initially restricted to Müller glia end feet at the ILM, but as the disease progresses this adduct appears in the radial fibers extending toward the outer retina. Moreover, our in vitro data clearly indicate that FDP-lysine is pathogenic for retinal Müller glia.

Measurements of specific products of lipid peroxidation have revealed that concentrations of MDA and 4-hydroxyalkenals are increased in retinas from STZ-diabetic rats as early as 6 weeks after the induction of diabetes [41-43]. Higher lipid peroxide levels have also been reported in retinas from human subjects with diabetes [44]. Despite these previous findings, we found no evidence to suggest that protein modifications by MDA, 4-HNE, or 4-HHE are significantly increased in the diabetic rat retina. The antibodies used in the current study have been comprehensively tested and validated in immunohistochemistry-based studies [31,45,46], and thus it seems unlikely that the apparent discrepancy in these results can be attributed to the loss or masking of relevant ALE epitopes. An alternative explanation could be that any increase in the generation of these adducts may be effectively compensated for by their rapid degradation via the ubiquitin–proteasomal pathway. Some support for this possibility comes from a previous study by Du et al. [47] who found that the increased formation of oxidatively modified proteins in retinal cells treated with high glucose was only detectable following inhibition of proteosomal activity.

An obvious question that arises from this study is: “Why do FDP-lysine adducts selectively accumulate in Müller glia during diabetes”? Although further studies are clearly needed to address this issue, some insights may be gleaned from the existing literature. In contrast to MDA and 4-hydroxyalkenals, ACR, the precursor of FDP-lysine, is not only formed endogenously during lipid peroxidation but can also be produced as a result of the oxidation of threonine by the myeloperoxidase system of neutrophils [48] and the amine-oxidase-mediated degradation of the polyamines spermine and spermidine [49]. One of the distinctive features of Müller glia that could make them particularly susceptible to ACR/FDP-lysine formation during diabetes is that these cells constitute the main polyamine storage cells of the retina [50]. As such, the selective accumulation of FDP-lysine-modified proteins in these cells could potentially be accounted for if polyamine catabolism were modified in the diabetic retina. Although future work is needed in this area, it has been reported that plasma amine oxidase activity is significantly elevated in patients with type 1 and type 2 diabetes [51,52] and correlates with the incidence of diabetic retinopathy [52]. While changes in polyamine catabolism could underlie the diabetes-induced accumulation of FDP-lysine in Müller glia, an alternative explanation could be that these adducts originate from an exogenous rather than endogenous source. Our in vitro data clearly demonstrate that Müller glia are capable of internalizing and accumulating FDP-lysine-modified protein, and the observation that these adducts first appear in the Müller glia end feet at the vitreoretinal interface indicates that the vitreous humor could represent a potential source.

Previous studies have demonstrated increased levels of FDP-lysine at sites of brain ischemia [53], in the retina of an experimental model of retinitis pigmentosa [54], in brain tissue from Alzheimer disease patients [55], in atherosclerotic lesions from human aorta [30], and in spinal cord following traumatic injury [56]. It is perhaps surprising, therefore, that no studies to date have examined the effects of FDP-lysine accumulation on cellular function and survival in any cell type. In the present study we have explored the role of FDP-lysine on diabetic retinopathy-linked pathology. This adduct causes Müller glia dysfunction, including increased expression of cytokines and dysregulated Kir4.1 expression. This may be directly linked to oxidative stress, as demonstrated by HO-1 induction concomitant with FDP-lysine-mediated loss of antioxidant defenses [39]. In addition, previous work has shown that ocular inflammation is associated with a downregulation of inwardly rectifying K^+^ currents in Müller cells of the rat retina [57]. Thus, it is also possible that the downregulation of the Kir4.1 channel observed in the present study may, at least in part, be related to the upregulation of inflammatory cytokines.

Diabetes is one of the few pathological states where Müller cell apoptosis has been shown to occur [12]. Nuclear translocation of glyceraldehyde-3-phosphate dehydrogenase (GAPDH) is thought to be a critical step in the diabetes-induced apoptosis of retinal Müller cells [58], and this mechanism is reported to play a significant role in the development and progression of diabetic retinopathy [59,60]. In the current study, exposure of MIO-M1 cells to a high concentration of FDP-lysine-HSA caused extensive apoptosis, suggesting that the observed accumulation of these adducts could potentially contribute to Müller cell death during long-term diabetes. Interestingly, FDP-lysine binds to and inactivates GAPDH [39], although whether this acts as stimulus for the nuclear translocation of this protein remains unknown. It would also be of interest to examine the effects of FDP-lysine treatment on the regulation of IL-1β and the E3 ubiquitin ligase SIAH-1, both of which have been implicated in the nuclear translocation and accumulation of GAPDH in retinal Müller cells under hyperglycemic conditions [61,62]. Exposure of MIO-M1 to 0.5 mg/ml FDP-lysine-HSA not only triggered widespread apoptosis but was also associated with a marked increase in the accumulation of FDP-lysine-modified protein. This is most likely explained by the fact that as apoptosis proceeds, the integrity of the plasma membrane is disrupted and cells become increasingly permeable to large macromolecules [63].

In conclusion this study has demonstrated that FDP-lysine accumulates in Müller glia during diabetes and that this adduct could be linked to dysfunction of these important retinal cells. Over the last few years, several pharmacological agents that display promising reactivity toward both ACR and FDP-lysine adducts have been identified [27]. Preclinical testing of these compounds in appropriate animal models of diabetes may provide a unique opportunity to begin to address the role of Müller cell changes in the pathogenesis of diabetic retinopathy.
